# The Use of Genetics for the Management of a Recovering Population: Temporal Assessment of Migratory Peregrine Falcons in North America

**DOI:** 10.1371/journal.pone.0014042

**Published:** 2010-11-18

**Authors:** Jeff A. Johnson, Sandra L. Talbot, George K. Sage, Kurt K. Burnham, Joseph W. Brown, Tom L. Maechtle, William S. Seegar, Michael A. Yates, Bud Anderson, David P. Mindell

**Affiliations:** 1 Department of Biological Sciences, Institute of Applied Sciences, University of North Texas, Denton, Texas, United States of America; 2 Alaska Science Center, U.S. Geological Survey, Anchorage, Alaska, United States of America; 3 High Arctic Institute, Orion, Illinois, United States of America; 4 Department of Biological Sciences, University of Idaho, Moscow, Idaho, United States of America; 5 Sheridan, Wyoming, United States of America; 6 Edgewood Research Development and Engineering Center, Department of Army, Aberdeen Proving Ground, Maryland, United States of America; 7 Raptor Research Center, Boise State University, Minden, Nevada, United States of America; 8 Falcon Research Group, Bow, Washington, United States of America; 9 California Academy of Sciences, San Francisco, California, United States of America; Smithsonian Institution National Zoological Park, United States of America

## Abstract

**Background:**

Our ability to monitor populations or species that were once threatened or endangered and in the process of recovery is enhanced by using genetic methods to assess overall population stability and size over time. This can be accomplished most directly by obtaining genetic measures from temporally-spaced samples that reflect the overall stability of the population as given by changes in genetic diversity levels (allelic richness and heterozygosity), degree of population differentiation (*F*_ST_ and *D*_EST_), and effective population size (*N*_e_). The primary goal of any recovery effort is to produce a long-term self-sustaining population, and these genetic measures provide a metric by which we can gauge our progress and help make important management decisions.

**Methodology/Principal Findings:**

The peregrine falcon in North America (*Falco peregrinus tundrius* and *anatum*) was delisted in 1994 and 1999, respectively, and its abundance will be monitored by the species Recovery Team every three years until 2015. Although the United States Fish and Wildlife Service makes a distinction between *tundrius* and *anatum* subspecies, our genetic results based on eleven microsatellite loci suggest limited differentiation that can be attributed to an isolation by distance relationship and warrant no delineation of these two subspecies in its northern latitudinal distribution from Alaska through Canada into Greenland. Using temporal samples collected at Padre Island, Texas during migration (seven temporal time periods between 1985–2007), no significant differences in genetic diversity or significant population differentiation in allele frequencies between time periods were observed and were indistinguishable from those obtained from *tundrius*/*anatum* breeding locations throughout their northern distribution. Estimates of harmonic mean *N*_e_ were variable and imprecise, but always greater than 500 when employing multiple temporal genetic methods.

**Conclusions/Significance:**

These results, including those from simulations to assess the power of each method to estimate *N*_e_, suggest a stable or growing population, which is consistent with ongoing field-based monitoring surveys. Therefore, historic and continuing efforts to prevent the extinction of the peregrine falcon in North America appear successful with no indication of recent decline, at least from the northern latitude range-wide perspective. The results also further highlight the importance of archiving samples and their use for continual assessment of population recovery and long-term viability.

## Introduction

In cases where populations or species have a recent history of decline followed by increase, the use of genetic data can be a powerful tool for monitoring progress in conservation efforts [1]–[4]. For example, estimates of the genetic diversity (allelic and heterozygosity), effective population size (*N*_e_), gene flow or dispersal, and population admixture can provide information useful for making future management decisions to prevent further population decline and extinction. For species previously considered threatened or endangered, section 4(g)(1) of the Endangered Species Act requires the U.S. Fish and Wildlife Service in cooperation with the States to monitor a species for a minimum of five years after being removed from the List of Endangered and Threatened Wildlife and Plants to ensure they maintain non-threatened status. The incorporation of genetic monitoring into such programs can provide information on the progress made in creating and maintaining a self-sustaining population, regardless if genetic measures were addressed in the original rulemaking or listing process [e.g., 5–7]. This approach can be particularly important with populations or species that have wide geographic distributions over challenging terrain (e.g., mountainous) where accurate demographic measures from the field are costly or difficult to obtain.

The peregrine falcon (*Falco peregrinus*) provides an example species recovery plan that could benefit from ongoing genetic monitoring. Globally, the peregrine falcon consists of nineteen subspecies and is found on every continent with the exception of Antarctica [8], [9]. In North America, three subspecies are currently recognized [10]. *F. p. pealei* is a year-round resident of the Pacific Northwest from the coasts of northern Washington and British Columbia extending to the Aleutian Islands in Alaska. *F. p. tundrius* breeds throughout the Arctic tundra of Alaska, Canada and western Greenland, and *F. p. anatum* breeds south of the tundra to northern Mexico, except in coastal areas in the Pacific Northwest. Both *F. p. tundrius* and *anatum* are migratory with *tundrius* wintering as far south as central Argentina and Chile in South America [11,12; see also 13].

Historic estimates of peregrine falcon abundance range from 400 to 500 pairs in Greenland [14], 1,000 to 3,500 [15] to 7,548 pairs [16] in the Arctic, and 7,000 to 10,000 total pairs in North America [17]. Following the late 1940s, many peregrine falcon populations suffered a steady decline due primarily to exposure from organochlorines, including DDT (1,1,1-trichloro-2,2-bis[p-chlorophenyl]-ethane) and its principle metabolite DDE (1,1-dichloro-2, 2-bis[p-chlorophenyl]-ethylene), which caused direct mortality or adversely affected their reproduction and egg production [18]–[21]. By 1964, peregrine falcons nesting east of the Rocky Mountains south of the boreal forests in Canada (*F. p. anatum*) were essentially extirpated [22], [23]. To the west in the Rocky Mountains, the number of peregrine falcons (*F. p. anatum*) was also significantly reduced with only 15 (29%) of 51 known historic nest sites occupied in 1964 [24], and by 1979 after a much more extensive survey of historic nest site locations, only 12 (7.5%) out of 160 were occupied covering an area of four million km^2^ [25]. Declines in the Arctic (*F. p. tundrius*) were less severe; however, it is estimated that their abundance was reduced approximately 50 to 60% by 1975 [20], [26]. For the Peale's falcon (*F. p. pealei*), abundance remained relatively stable during this time period [26], presumably due to their specialized diet feeding predominately on sea birds (e.g., alcids; [27], [28] as opposed to terrestrial avian prey that were more likely to be exposed to DDT.

In the past two decades, peregrine falcons in the U.S. and Canada, including Europe [8], [29], have made a remarkable recovery due to the ban of DDT in 1969 and 1972 in Canada and the U.S., respectively, the inclusion of *F. p. anatum* and *F. p. tundrius* on the precursor of the federal Endangered Species List in 1970 (35 FR 16047), and extensive propagation and release efforts made by many conservation groups [30]. As a result, the species was delisted in 1994 (*F. p. tundrius*; 59 FR 50796), and 1999 (*F. p. anatum*; 64 FR 46541-46558) in the U. S., and reassigned Special Concern status in April 2007 in Canada. Current estimate of total breeding population size for both *F. p. tundrius* and *F. p. anatum* is between 4,300 and 10,400 [31]; considering immature and floater (non-breeders) individuals, the total population size could be between 40,000 to 50,000 [13]. These estimates are based on both direct and indirect counts, including projections made from available potential nesting sites. Because our current estimates of population size are imprecise, significant uncertainty exists for making decisions for management purposes.

The primary aim of this study was to determine the overall stability and effective size of high latitude peregrine falcon populations in North American and Greenland, *F. p. anatum* and *tundrius*. This was accomplished using temporally segregated samples from both migratory and breeding peregrine falcons sampled during fall and spring migration through Padre Island, Texas over a 21 year period (∼7 generations) and from southwestern Greenland over a 12–14 year period (∼4 generations), respectively. We assume that the sampled migratory individuals from Padre Island used in this study possess high latitude breeding distributions throughout Alaska, Canada and Greenland as supported by band recovery [31], satellite telemetry studies [12], [32], [33] and genetics (*this study*; see below), and therefore represent the northern peregrine falcon breeding population in North America. Our temporal sampling allowed the investigation of overall population stability by assessing allele frequency change over time and the estimation of *N*_e_ for both the migrant population and a focal breeding population in Greenland. These results are useful for conservation monitoring purposes and for making decisions that may influence future peregrine falcon population viability in North America.

## Methods

### Sampling and DNA extraction

**Figure 1:**
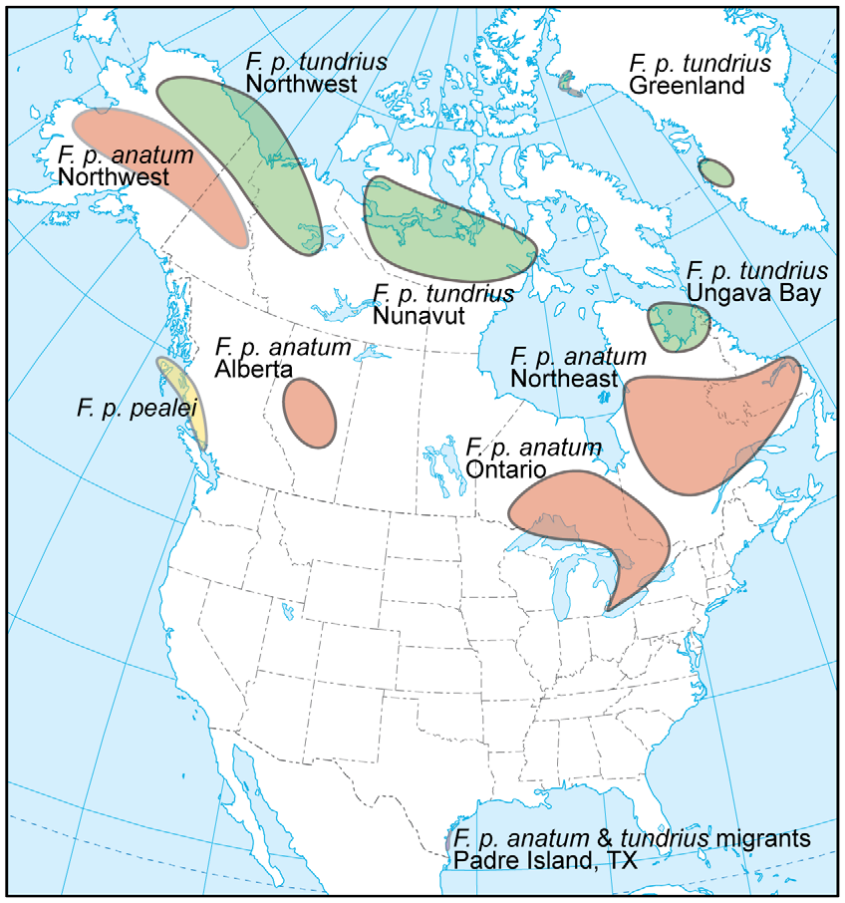
Peregrine falcon population sampling locations in North America. Samples sizes for each area are given in Table 1.

All samples were obtained from birds caught and bled under government permits, and all birds were released after processing. A total of 292 peregrine falcons were sampled for genetic analyses during migration through Padre Island, Texas. Blood samples were taken during both autumn and spring migration for each of the following temporal subsets: 1985/86, 1988/89, and 2006/07, with additional samples from spring 2001 migration period (see Table 1 for sample sizes). All samples were kept frozen, and DNA extractions were performed using methods described elsewhere [34]. An additional 349 samples were obtained from the contemporary northern breeding distribution of peregrine falcons throughout Alaska, Canada and western Greenland, of which 168 samples were used in a previous study [35]. The samples collected from breeding territories were included in this study to verify the degree of population subdivision throughout their northern breeding distribution from which the Padre Island migrants likely originated. For analysis purposes and adequate sample size considerations, individuals were grouped into geographic sampling regions for each of the subspecies (see Fig. 1; Table 1). Two additional subspecies, *F. p. cassini* (n = 25) from South America and *F. p. macropus* (n = 15) from Australia, were included in the analyses for comparative purposes.

**Table 1:** Levels of nuclear microsatellite genetic diversity (11 loci) for regional peregrine falcon sample locations.

	*N*	*A*	*AR*	*H*_O_	*H*_e_	*F*_is_ 7
***F. p. pealei***	33	4.1 (0.6)	3.7 (0.5)	0.489 (0.084)	0.503 (0.086)	0.037
***F. p. tundrius***						
Northwest^1^	20	4.5 (0.8)	4.1 (0.7)	0.425 (0.075)	0.525 (0.086)	0.194*
Nunavut	41	5.2 (0.8)	4.4 (0.6)	0.525 (0.077)	0.549 (0.074)	0.044
Ungava Bay	13	4.0 (0.7)	4.0 (0.7)	0.455 (0.099)	0.485 (0.092)	0.065
Greenland 1990^2^	37	5.5 (0.8)	4.4 (0.6)	0.490 (0.105)	0.536 (0.087)	0.105*
Greenland 2001-04^3^	42	5.5 (1.0)	4.6 (0.7)	0.474 (0.081)	0.528 (0.086)	0.088*
***F. p. anatum***						
Northwest^4^	76	5.3 (0.9)	4.1 (0.6)	0.481 (0.092)	0.506 (0.086)	0.050*
Alberta	21	4.2 (0.6)	3.9 (0.5)	0.524 (0.086)	0.537 (0.079)	0.025
Ontario	47	5.1 (0.8)	4.3 (0.7)	0.569 (0.093)	0.535 (0.083)	−0.064
Northeast^5^	19	4.5 (0.7)	4.2 (0.7)	0.536 (0.088)	0.557 (0.083)	0.039
***F. p. tundrius*** ** and ** ***anatum*** ** migrants (Padre Island, TX)**						
Fall 1985	46	5.0 (0.9)	4.4 (0.7)	0.458 (0.089)	0.526 (0.087)	0.131*
Spring 1986	46	5.1 (0.9)	4.3 (0.7)	0.518 (0.082)	0.542 (0.085)	0.046
Fall 1988	46	4.9 (0.8)	4.1 (0.5)	0.518 (0.081)	0.526 (0.074)	0.016
Spring 1989	46	5.3 (0.9)	4.2 (0.6)	0.522 (0.090)	0.529 (0.088)	0.013
Spring 2001	42	4.9 (0.9)	4.1 (0.6)	0.479 (0.084)	0.522 (0.084)	0.085
Fall 2006	36	5.1 (0.9)	4.2 (0.7)	0.453 (0.079)	0.525 (0.083)	0.139
Spring 2007	30	4.7 (0.8)	4.2 (0.6)	0.458 (0.074)	0.509 (0.078)	0.103
***F. p. cassini*** 6	25	2.5 (0.3)	2.4 (0.3)	0.373 (0.063)	0.389 (0.062)	0.042
***F. p. macropus***	15	2.5 (0.5)	2.4 (0.5)	0.289 (0.092)	0.283 (0.086)	−0.023

*N*, samples size; *A*, mean number of alleles; *AR*, allelic richness; *H*_O_, observed heterozygosity; *H*_e_, expected heterozygosity. Standard error is given in parentheses.

1 Colville River, Alaska; Horton River, Alaska; Mackenzie Valley, NWT, Canada.

2 Kangerlussuaq, Greenland.

3 Thule (n = 15) and Kangerlussuaq (n = 27) sampled 2001-04.

4 Yukon River, Alaska; Tanana River, Alaska; Porcupine River, Alaska; Yukon, Canada.

5 Quebec, Newfoundland, and Labrador, Canada.

6 Patagonia, Argentina.

7 significant *F*_is_ indicated by

*(p<0.003).

### Genotyping

Eleven microsatellite loci originally developed for the peregrine falcon (Fp5, Fp13, Fp31, Fp46-1, Fp54, Fp79-4, Fp82-2, Fp86-2, Fp89, Fp92-1, Fp107; [36]) were used for the microsatellite analyses. All microsatellite loci were dinucleotide repeats, and protocols used for PCR amplification have been described elsewhere [34], [35], [37]. Genotypic data generated in different laboratories using different procedures were calibrated using a subset of samples (n≥4) for each of the eleven microsatellite loci. No ambiguities were observed across all loci after calibration.

### Statistical analyses

#### Genetic diversity

Microsatellite genotypes were tested for linkage equilibrium and departure from Hardy-Weinberg equilibrium within each population at each locus using the computer program GDA [38]. Sequential Bonferroni corrections were applied to correct for multiple simultaneous comparisons [39]. Mean number of alleles per locus (*A*) and observed (*H*_o_) and expected (*H*_e_) heterozygosity values were calculated using GDA. Measures of allelic richness (*AR*) were calculated using the program Fstat version 2.9.3.2 [40]. *AR* estimates control for uneven sample sizes among populations [41]. Differences in microsatellite genetic diversity estimates between sample locations and time periods were tested for significance using a Wilcoxon signed-rank test. Measures of *F*_IS_ and its significance for each sampled population was calculated using Fisher's exact test within Genepop v. 4.0.10 ([42], [43]; http://genepop.curtin.edu.au) after adjusting the *p*-value to account for multiple simultaneous comparisons [39].

#### Population subdivision

The degree of population subdivision between sample locations and temporal sampling periods was investigated using the Bayesian method of Pritchard et al. [44] and Falush et al. [45], implemented in the program Structure version 2.1. The number of genetically distinct clusters (*K*), or populations, was identified based on allele frequencies across loci while minimizing linkage and violations to Hardy-Weinberg equilibrium. The most likely value of *K* is determined by comparing the likelihood of the data for different values of *K*. To determine the number of clusters, we also calculated the rate of change in the log probability of the data between successive K values (Δ*K*) plotted against *K* following Evanno et al. [46]. Analyses were performed with all samples and subspecies, including additional analysis without *F. p. pealei*, *F. p. cassini* and *F. p. macropus* samples to determine the influence the latter three subspecies have on the overall approximation of *K*. Calculations were conducted with a burn-in period of 10^5^ iterations followed by an additional 10^6^ iterations. Each simulation from *K* = 1 to 8 was performed four times using an ancestry model allowing admixture where individual α was inferred from the data for each cluster (alpha >1 means that most individuals are admixed; [45]), and a model of correlated allele frequencies that did not include prior information on sampling origin. Final results from Structure were visualized using the program Distruct [47]. The degree of population subdivision was also explored as implemented in the software TESS [48]. This latter approach determines the number of groups similar to Structure, but differs by taking into account the spatial organization of individuals and incorporates a regularization procedure that helps facilitate the choice of *K* [48]–[50]. The method implemented in TESS is also less influenced by Isolation by Distance compared to methods such as Structure when identifying the number of distinct clusters when clinal variation exists [50], [51]. The MCMC algorithm was run under admixture model with interaction parameter Ψ = 0.7, with 10,000 burn-in and 50,000 sweeps. Twenty independent iterations were run for *K* = 2–7 and after identifying the value of *K* that produced the highest likelihood, this was run 100 times and the 20 highest likelihood runs for *K*_max_ were averaged using CLUMPP version 1.1.2 [52] applying the Full Search algorithm and the G' pairwise matrix similarity statistics.

Estimates of genetic differentiation based on pairwise *F*_ST_ and *D*_est_ values were also obtained to further investigate the overall stability between sampling locations and temporal sampling periods. *F*_ST_ values were calculated following Weir & Cockerham [53] as implemented in Arlequin version 3.11 [54], and *D*_est_ values [55] were calculated using Spade [56] and bootstrap proportions for estimates of 95% confidence intervals (CI) were based on 1,000 permutations. The program Isolation By Distance Web Service (IBDWS), version 3.16 [57] was used to perform a Mantel test with 10,000 randomizations to examine the correlation between matrices of genetic distance (pairwise *F*_ST_ and *D*_est_) and geographic distance of breeding sample locations of *anatum* and *tundrius*. A second set of analyses that included *pealei* samples were also performed. Geographic distance between sample locations was measured as the euclidean distance (km) using the ruler implemented in Google Earth version 5.2.1.1329.

#### Effective population size

Genetic estimates of effective population size (*N*_e_) were calculated using different methodological approaches to assess the robustness of our results. Two general approaches to estimating *N*_e_ were explored: 1) analyzing single time period population samples, and 2) analyzing multiple temporal samples from the same population. Although the temporal approach has been shown to outperform single-sample methods when assuming a closed population [1], [58], [59], recent analytical developments have improved both the precision and accuracy of *N*_e_ estimates from individual population samples [e.g., 60,61]. We used a method originally based on linkage disequilibrium (LD; [62]) to estimate contemporary *N*_e_ from individual sampling periods. The LD method included a bias correction [60] as implemented in the program LDNe [63], which has been shown to improve performance even with non-ideal populations (e.g. skewed sex ratios or non-random variance in reproductive success; [60]). Estimates of *N*_e_ were obtained for each of the spring migratory sampling periods collected from Padre Island, TX. We do not use this method to estimate *N*_e_ for any of the breeding populations because we cannot assume a closed population [64], [65]. A jackknife method was used to obtain 95% confidence intervals (CI) on loci, and estimates were calculated assuming random mating and excluded all alleles ≤0.01 [63].

To estimate *N*_e_ based on multiple sampling periods for the migrant population from Padre Island, we employed three methods that are based on the premise that temporal variance in neutral genetic allele frequencies is inversely proportional to *N*_e_ due to the effects of genetic drift in the absence of migration and mutation [66], [67]. The first method is based on the standardized variance of change in allele frequencies (*F*_k_) between at least two sampling periods (equation 11, [68]; see also [69]). Because bias can exist with this method when estimates are based on small sample sizes and skewed allele frequencies [70], we used the weighing scheme of Jorde & Ryman [71] to provide an estimate of *N*_e_ with our dataset. Using sampling plan I [68], estimates of *N*_e_ and 95% confidence intervals were obtained using the program TempoFs [71]. Sampling plan I (i.e. nondestructive sampling) requires an estimate of population census size (*N*) to calculate *N*_e_. Because we do not have a precise estimate of *N* for the peregrine falcon population, we calculated *N*_e_ using a range of values from 1,000 to 100,000 individuals to determine if uncertainty in *N* influences our estimate of *N*_e_. Two additional estimates of *N*_e_ were obtained for the Padre Island migrant population using a coalescent-based method as employed in the program TM3 [72] and a pseudo-likelihood method implemented in the program MLNE 2.3 [65], [73]. Both methods were used to calculate *N*_e_ of the migrant population while assuming an *N*_e-MAX_ of 10,000, no immigration, and a generation time of three years.

To estimate *N*_e_ based on multiple temporal sampling periods for the Greenland population, we used MLNE 2.3 while assuming 1) a closed population (*N*_eCLOSED_), and 2) accounting for immigration from a potential source population (*N*_eOPEN_). Estimates of *N*_e_ from temporal data when mistakenly assuming a closed population are likely to be incorrect because, in addition to the effects of genetic drift, immigration will influence allele frequencies of the population to an extent that is related to the amount of differentiation between populations [59], [64], [65], [74]. Therefore, when immigration is present, methods that account for this effect should be employed to generate accurate estimates of *N*_e_. Using MLNE, two temporally spaced datasets from Greenland (1990 and 2001-04; four generations) were used to estimate both *N*_eCLOSED_ and *N*_eOPEN_ to assess the potential influence of immigration on our estimate of *N*_e_ and for comparative purposes with our estimates from the migrant Padre Island population *N*_e_. Similar to the migrant dataset, 10,000 was used as our *N*_eMAX_. We used the pooled allele frequencies from contemporary *tundrius* and *anatum* breeding locations as the potential source population for immigrants into Greenland for estimating *N*_eOPEN_. Additional estimates were also calculated using the spring migrants from Padre Island, TX as the source population to evaluate the choice of source population on *N*_eOPEN_.

#### Simulations

To assess the utility and precision of methods used to estimate *N*_e_, we used simulated data representing multiple populations of specified size. This was done primarily to determine our ability to estimate *N*_e_ in populations of large size where drift is not likely to play a strong role influencing allele frequency change over a short time periods, e.g., seven generations (as with this study). Using the empirical data from spring 1986 as our initial sampling period (*T*_0_), we used the program BottleSim [75] to simulate seven generations (*T*_7_) at population sizes *N*_e_ = 50, 100, 200, 300, 500, 1000, 2000, and 5000. For each simulation based on 1000 iterations, we used the settings for maximum generation overlap (100%), random mating, three years for age at first breeding, 12-year longevity, and equal sex ratios [13]. Estimates of *N*_e_ were calculated similar to the empirical data, and their deviations from the specified *N*_e_ were then determined and directly compared to results obtained using the migrant Padre, TX temporal dataset. We also assessed levels of differentiation among ten populations from each of the simulated datasets of known size using similar samples sizes (n = 46). This was done to investigate the development of genetic differentiation relative to *N*_e_ after seven generations had passed similar to our empirical dataset.

## Results

### Genetic diversity measures

Eight of the eleven microsatellite loci were polymorphic in all peregrine falcon sampling locations. Locus Fp5 was monomorphic in *F. p. tundrius* Northwest and Ungava Bay, *F. p. anatum* Alberta and Northeast, *F. p. cassini*, and Padre Island spring 2001 migrant sampling locations. Loci Fp54 and Fp92-1were both monomorphic for *F. p. macropus*. After adjusting for multiple comparisons, significant departures from Hardy-Weinberg equilibrium in the form of heterozygote deficiencies were observed in one locus (Fp92-1) among four sampling locations (Northwest and 1990 Greenland, *F. p. tundrius*; Northwest, *F. p. anatum*; Padre Island fall migrants 1985). Similarly, significant *F*_IS_ values were observed with five sampled locations (Table 1; heterozygote deficit), and three (Greenland 1990 & 2001-04, *F. p. tundrius*; Northwest, *F. p. anatum*) of the five remained significant after excluding locus Fp92-1 from the analysis. No pairwise comparisons testing for linkage disequilibrium were significant after correcting for multiple comparisons.

The majority of microsatellite genetic diversity estimates do not differ significantly (Wilcoxon signed-rank test, *p*>0.05) between geographic sampling locations in North America or between temporal sampling periods from Padre Island, Texas or Greenland (Table 1). Allelic richness (AR) varied from 3.7±0.5 (± s.e.) alleles per locus in *F. p. pealei* to 4.6±0.7 alleles per locus in *F. p. tundrius* from western Greenland. The few cases for *F. p. pealei* possessed significantly lower AR compared to the *tundrius* populations in Nunavut (*Z* = −2.667; *p* = 0.008) and Greenland (*Z* = −2.667; *p* = 0.008) and the *anatum* population in Ontario (*Z* = −2.134; *p* = 0.033). Expected heterozygosity (*H*_e_) ranged from 0.485±0.092 in Ungava Bay *F. p. tundrius* from northeastern Canada to 0.557±0.083 in *F. p. anatum* from eastern Canada. A significant difference in *H*_e_ was observed between *F. p. tundrius* populations in Ungava Bay and Nunavut (*Z* = −2.223; *p* = 0.026). Genetic diversity estimates for *F. p. cassini* and *macropus* from Argentina and Australia, respectively, were significantly lower in all comparisons of AR when compared to all sampling locations for the North American subspecies *anatum*, *tundrius*, and *pealei* (*p*<0.02); Table 1). *F. p. macropus H*_e_ was significantly lower than *H*_e_ estimates from all *anatum*, *tundrius*, and *pealei* populations (*p*<0.04), with the exception of Ungava Bay *F. p. tundrius* (*Z* = −1.778; *p* = 0.075). *F. p. cassini H*_e_ was not significantly different (*p*>0.06) from any of the *H*_e_ estimates from *anatum*, *tundrius*, and *pealei* populations.

### Population structure

**Figure 2:**
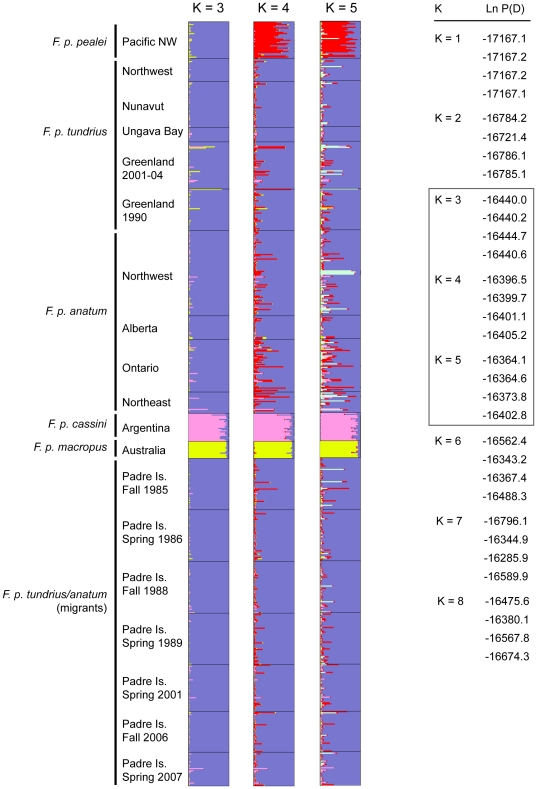
Results from Structure analysis for all sampled peregrine falcon populations. (A) Assignment of individuals to K = 3 to 5 inferred clusters based on 11 microsatellite loci. Colors indicate different inferred clusters and their magnitude represents the posterior probability that the individual belongs to a particular cluster. (B) Estimated log probability values [Ln (P(D)] for each run for *K* = 1 to 8. The box highlighting *K* = 3 to 5 indicates the lowest values of *K* with the highest likelihood values. These results remained similar after excluding *F. p. cassini* and *macropus* from the analysis (data not shown).

The posterior probability values for each value of *K* with Structure, while using the complete dataset that included *F. p. cassini* and *macropus*, plateau at *K* = 3 to *K* = 5 with Ln P(D) values from multiple runs at *K* = 3 (SD = 2.2) and *K* = 4 (SD = 3.6) being more consistent across runs compared to *K* = 5 (SD = 18.2; Fig. 2). When we used Δ*K* to infer the number of clusters, *K* = 3 was clearly inferred for the complete dataset using all sampled subspecies. Results from Structure when using only data from North American subspecies (*F. p. pealei*, *tundrius*, *anatum*) indicated the highest posterior probability values for *K* = 2 (Ln P(D) = −15843.5), while *K* = 1 and *K* = 3 had lower posterior probability values (−15886.7 and −15937.6, respectively). We are unable to evaluate between *K* = 1 and *K* = 2 using the Δ*K* method (see Evanno et al. 2005). For *K* = 2 in this second analysis, *F. p. tundrius* and *anatum* sample locations possessed a relatively high proportion of membership to the same inferred cluster (0.870 to 0.959 and 0.759 to 0.922, respectively) and the majority of the *pealei* samples were assigned to the second cluster at a lower proportion (proportion of membership  = 0.661). Samples collected from Padre Island across all years clustered with high support (>0.924; data not shown) with samples collected from breeding grounds identified as *F. p. tundrius/anatum*.

The results from TESS corroborated those found with Structure. The minimum DIC value (30669) was achieved with *K* = 4 (*K* = 2, DIC value  = 31736; *K* = 3, DIC value  = 31163) and plateaus with higher values of *K*. The average value for the 20% best runs when *K* = 4 showed that the *tundrius* and *anatum* individuals clustered in a single group, which also included all of the migrant samples collected from Padre Island, whereas the remaining three subspecies, *pealei*, *macropus* and *cassini*, were in separate clusters each with high support (data not shown).

Estimates of *F*_ST_ and *D*_est_ largely agreed with the results from Structure and TESS, indicating strong genetic differentiation between subspecies with the exception of those comparisons between *F. p. tundrius* and *anatum* which showed much lower levels of differentiation (Table S1). Although 16 of the 20 pairwise *F*_ST_ comparisons were significant (*p*<0.001) after correction for multiple comparisons between *F. p. tundrius* and *anatum* sampled breeding territories, the values were low (*F*_ST_ = 0.006 to 0.050) and similar in magnitude to those obtained from within subspecies comparisons (*F*_ST_ = 0.007 to 0.026 and 0.016 to 0.033 for *tundrius* and *anatum*, respectively) of which some were also significant (*p*<0.001; see Table S1). Estimates of *D*_est_ between *F. p. tundrius* and *anatum* were also low (*D*_est_ = 0.000 to 0.032), with only one comparison being significantly different from zero (Ontario & Greenland_1990). *D*_est_ values between *F. p. tundrius* and *anatum* were similar to those obtained from pairwise comparisons between sample locations within *F. p. anatum* (*D*_est_ = 0.003 to 0.029), while pairwise comparisons between *tundrius* subspecies locations were consistently low (*D*_est_ = 0.000 to 0.012) and not significantly different from zero (Table S1). After excluding samples from Ungava Bay due to low population sample size (n = 15), significant isolation by distance was observed among breeding sample locations of *F. p. tundrius* and *anatum* using both pairwise *F*_ST_ (*r* = 0.663; Mantel test *P* = 0.003) and *D*_est_ (*r* = 0.649; Mantel test *P* = 0.001) measures. Similarly, significant isolation by distance was observed among *F. p. tundrius* and *anatum* sample locations when southwest Ontario samples were excluded from the analyses (*F*_ST_, *r* = 0.668, Mantel test *P* = 0.012; *D*_EST_, *r* = 0.579, Mantel test *P* = 0.035). However, isolation by distance was not supported when we included *F. p. pealei* genetic distance measures with *tundrius* and *anatum* breeding locations (*F*_ST_, *r* = 0.092, Mantel test *P* = 0.324; *D*_est_, *r* = 0.113, Mantel test *P* = 0.288).

When comparing migrant peregrines from Padre Island with samples collected on breeding territories, far fewer pairwise *F*_ST_ and *D*_est_ comparisons were significant with those made with *F. p. tundrius* (23% and 0% out of 35 comparisons, respectively) than with *anatum* samples (71% and 14% out of 28 comparisons, respectively; Table S1). No pairwise *F*_ST_ or *D*_est_ comparisons between migrant Padre Island temporal samples were significant across sampling periods, indicating stable allele frequency distributions over a 22-year period. The simulated datasets of known size at *N*_e_ of ≤300 following seven generations were all significantly different from each other based on allele frequency distributions after sequential Bonferroni correction; whereas at *N*_e_ of 500, eight of forty-five comparisons were significant and at *N*_e_ of ≥1000, none of the pairwise comparisons were significant (data not shown).

### Effective population size

Point estimates of *N*_e_ for the migrant peregrine falcon population varied depending on the choice of method, but in all cases, the values were high and ranged from 509 to >10,000 breeding individuals (Table 2). Reported 95% confidence levels around each point estimates were wide, with all of cases, regardless of method, extending to infinity, or at least the maximum allowable value (>10,000) used in each of the analyses (i.e., *N*_e-MAX_). The choice of the census population size (*N*, 1,000 to 100,000; see Methods) used with the method implemented in the program TempoFs did not substantially influence our estimate of *N*_e_. For example, *N*_e_ was 450 (117-infinity) and 516 (121-infinity) using an *N* of 1,000 or 100,000, respectively. Estimates of *N*_e_ from the method LDNe using spring migratory single time periods ranged from 187.8 in the 2001 dataset and >10,000 in the remaining three periods (1986, 1989, and 2007) with 95% CIs ranging between 59.5 to infinity.

**Table 2:** Estimates of *N*_e_ for the migrant peregrine falcon population at Padre Island, TX.

	Number of	Method used to estimate *N*_e_			
Population	generations	LDNe^1^	TempFs	TM3	MLNE
Padre-spring	7	infinity	509.0	564.9	584.4
		(69.8-infinity)	(121.0-infinity)	(54.7-infinity)	(170.2-infinity)
Padre-fall	7	4294.0	864.0	6814.5	1278.3
		(82.7-infinity)	(201.0-infinity)	(211.2-infinity)	(240.3-infinity)

95% confidence intervals are provided in parentheses below each point estimate. Values indicated as “infinity” represent >10,000 breeding individuals (*N*_e-MAX_ of 10,000).

1 Estimates of *N*_e_ from LDNe are based on a single time period (i.e., the 7^th^ generation at the particular population size).

MLNE analyses on the Greenland population while allowing for immigration (see Methods) produced an *N*_eOPEN_ estimate of 122.7 (95% CI 55.3-590.7) with a joint migration estimate of 0.103 (95% CI 0.024-0.234). The choice of source population did not substantially affect our estimates of *N*_eOPEN_. When we defined the source population for potential immigrants as Padre Spring 2007, our estimates of *N*_eOPEN_ was 102.5 (95% CI 44.7-470.7) with a joint migration estimate of 0.221 (95% CI 0.056-0.734). Estimate of *N*_eCLOSED_ for the Greenland peregrine falcon population was 158.9 (95% CI 75.7-804.5).

**Figure 3:**
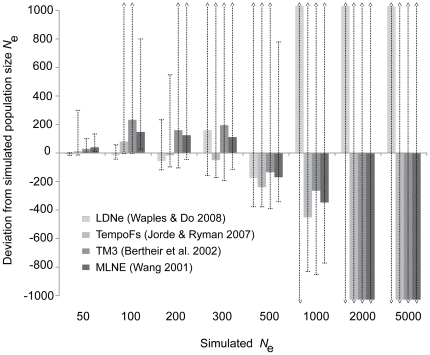
Estimates of *N*_e_ from simulated populations of known size. Three different temporal methods (TempoFs, TM3, MLNE) and a fourth method (LDNe) based on a single time period were used to estimate *N*_e_. Similar to our empirical data from Padre Island, TX, temporal estimates were based on seven generations (*T*_0_ - *T*_7_), while the single time period estimate is from the simulated 7^th^ generation (see methods). Bars represent deviated estimates of *N*_e_ from simulated population size and the dotted vertical lines reflect 95% confidence intervals. ^ =  values beyond the range of y-axis (see also Table 3 for point estimates used in this figure).

Results from our simulated datasets of known size ranging from 50 to 5,000 breeding individuals further supported our empirical data suggesting that the migrant population is of large size. As population size increased across simulations, the accuracy and precision of each method for calculating *N*_e_ decreased (Fig. 3; Table 3). Above *N*_e_ of 500, for example, point estimates from all methods differed from the actual simulated size by more than 200 individuals with wide 95% confidence intervals (Fig. 3). Interestingly, point count estimates of *N*_e_ at levels below 500 were more often overestimated, while ≥500 tended to be underestimated. The one obvious exception was with LDNe where estimates of *N*_e_≥1,000 were overestimated (Fig. 3; Table 3). These results suggest that our estimate of the migrant peregrine falcon population *N*_e_ is at least 500 and possibly >1000 breeding individuals.

**Table 3:** Estimates of *N*_e_ from simulated populations of known size.

Population	Number of	Method used to estimate *N*_e_			
size	generations	LDNe^1^	TempFs	TM3	MLNE
50	7	42.0	63.0	78.8	89.9
		(31.1–60.1)	(35.0–338.0)	(55.7–122.8)	(59.7–143.6)
100	7	87.6	180.0	333	248.3
		(56.7–168.8)	(96.0–1378.0)	(95.8–1169.9)	(126.9–751.1)
200	7	145.8	184.0	360.3	324.7
		(81.6–485.3)	(104.0–763.0)	(96.0–1825.1)	(155.0–1430.0)
300	7	463.3	251.0	495.1	411.4
		(143.4-infinity)	(128.0-infinity)	(105.5–6744.7)	(185.3–3614.7)
500	7	327.1	261.0	365	330.4
		(122.0-infinity)	(124.0-infinity)	(109.5–1770.2)	(159.2–1448.3)
1,000	7	infinity	549.0	735.5	653.1
		(0-infinity)	(169.0-infinity)	(149.5-infinity)	(225.5-infinity)
2,000	7	3742	953.0	616.3	605.4
		(197.2-infinity)	(296.0-infinity)	(117.4-infinity)	(223.2-infinity)
5,000	7	infinity	439.0	893.7	802.4
		(205.6-infinity)	(152.0-infinity)	(167.3-infinity)	(241.7-infinity)

95% confidence intervals are provided in parentheses below each point estimate. Values indicated as “infinity” represent >10,000 breeding individuals (*N*_e-MAX_ of 10,000).

1 Estimates of *N*_e_ from LDNe are based on a single time period (i.e., the 7th generation at the particular population size).

## Discussion

Population genetic data are a valuable tool for monitoring populations and species [e.g., 2–4,7], particularly those in the process of recovery. The peregrine falcon in North America is one such species that has required extensive monitoring to assess its progress toward achieving a sustainable population as defined by a federally mandated monitoring plan [76] subsequent to the species' delisting from endangered status in 1999 (64 FR 46541-46558). Here, we have utilized genetic data to assess the stability of the migrant peregrine falcon population in North America. Our results based on multiple methodological approaches indicate that this species is stable from a population genetics perspective as documented for breeding territories sampled throughout its entire northern latitudinal distribution including its migratory population at Padre Island, Texas.

### Assessment of population structure – *F. p. tundrius and anatum*

To support our use of peregrine falcon samples collected during migration at Padre Island to assess the species' northern high latitude population genetic stability, it was important to document levels of genetic differentiation among all presumed northern breeding areas that contribute to the migrant population. In a previous study investigating the genetic structure of peregrine falcon sampling locations across Canada, Brown et al. [35] documented no differentiation based on *F*_ST_ and Structure analyses between *F. p. tundrius* and *anatum* using samples collected prior to their population decline in the 1950s. These results suggested that a continuous phenotypic cline existed based on the subtle morphological and behavioral characters used originally to describe the separation of the two subspecies (e.g., [77]). In contrast, however, Brown et al. [35] did document low but significant differentiation between the two subspecies when using contemporary samples. This was particularly the case with sample locations from southeastern Ontario where stronger allele frequency fluctuations and allelic introgression from captive released birds were more likely to have occurred as compared to their sampling locations further north and to the west (see also [78], [79]; Fig. 2; Table S1).

Nearly 7,000 peregrine falcons were released in the United States and Canada between the years 1974 and 1999 [13]. Many of these birds originated from breeding stock that included non-native peregrine subspecies such as *peregrinus* and *brookei* from Europe, *cassini* from South America, and *macropus* from Australia, but to a lesser extent than individuals with either *anatum*, *tundrius* or *pealei* pure or mixed-ancestry [79]–[81]. Because peregrine falcons were extirpated throughout southeastern Canada and the United States east of the Rocky Mountains, the peregrine falcons that now reside in these areas are largely the result of the release programs [e.g., 82]. Therefore, it is not surprising that contemporary low levels of genetic differentiation were observed between peregrine falcon populations sampled in geographic areas that have been reestablished with captive-bred individuals, while no genetic differentiation was observed prior to the decline. Other release and supplementation programs have also documented similar effects [e.g., 83–86]. For example, Jacobsen et al. [29] reported significant microsatellite allele frequency changes before and after the reintroduction project of peregrine falcons *(F. p. peregrinus*) in southern Scandinavia [87].

Using *F. p. tundrius* and *anatum* samples from Brown et al. ([35]; n = 140) with additional sampling (n = 176) of both subspecies from previously unsampled geographic areas in Alaska (*tundrius* and *anatum*) and Greenland (*tundrius*), our results indicate little if no genetic population differentiation (Structure, TESS, *F*_ST_ and *D*_est_) among sampling locations of these two subspecies. The significant values that were observed were those comparisons that included *F. p. anatum* samples from eastern Canada or geographically distant locations, including three other subspecies (Fig. 2; Table S1). A significant Mantel correlation between genetic (*F*_ST_) and geographic distance matrices suggests an isolation by distance model of population differentiation (or regional equilibrium; [88]) throughout the high-latitude breeding distribution of *tundrius* and *anatum*. These results were also supported by a new measure (i.e., *D*_est_, [55]) that is useful for investigating population differentiation between geographic locations when using highly polymorphic markers such as microsatellite DNA [see also 89–91]. In this case, only a few pairwise *tundrius*/*anatum* or *anatum*/*anatum* comparisons were significant, all of which were those comparisons made with the Ontario sampled location (Table S1), and similar to *F*_ST_, an isolation by distance model of population structure could not be rejected based on *D*_est_. This is consistent with there being considerable gene flow among the populations considered as *tundrius* or *anatum* in Alaska, Canada and Greenland, and suggests that enough time may have passed for localized gene flow and genetic drift to stabilize and produce a pattern characteristic of migration-drift equilibrium throughout their high-latitude distribution (e.g., [88]). In contrast, all pairwise comparisons (*F*_ST_ and *D*_est_) that included other subspecies *pealei*, *cassini* and *macropus* were significant.

Although the degree of differentiation between *pealei* and *tundrius/anatum* was not as strong with Structure and TESS compared to *F*_ST_ and *D*_est_, the lower proportion of membership for a few individuals from the two groups was likely due to *pealei* also being used in the captive breeding program for which some post release individuals subsequently possessed mixed ancestry [79–81; see Figure 2]. Similarly, a few of the individuals collected as *pealei* may have been misidentified in the field where the subspecies' distribution overlaps with *anatum*. Although, *F. p. pealei* are non-migratory and they occupy coastal territories in the Pacific Northwest, extensive plumage variation in immature individuals exists, particularly in the southern portion of their range [13] where our *pealei* samples originated. No significant isolation by distance was observed after including the *F. p. pealei* sample location with *F. p. tundrius*/*anatum* breeding territories further suggesting no contemporary gene flow between *pealei* and *tundrius*/*anatum* populations. Therefore, the Structure results were likely influenced by introgression of alleles from individuals used in the release program [e.g., 29] and/or the lack of power to detect population structure in cases with relatively low levels of differentiation (e.g., *F*_ST_<0.03; [92], [93]).

With the exception of those comparisons made between high latitude populations of *F. p. tundrius* (see below), we remain cautious and resist equating reported population differentiation estimates with actual relative degree of differentiation between populations [e.g., 55,94–96]. Peregrine falcons in North America, particularly those on the East Coast and Midwest in both Canada and the U.S., have experienced dramatic changes in population abundance over the past half-century [13], [17], [30]. These lower latitude populations are unlikely to have reached the equilibrium conditions necessary for a direct interpretation of measures of differentiation. Additional work is necessary to explore how these measures are influenced by data from populations with differing demographic histories, particularly those that experienced recent decline followed by a rapid recovery.

Our results from Structure, TESS, *F*_ST_ and *D*_est_ do indicate, however, no genetic differentiation between *F. p. tundrius* and high latitude *anatum* sample locations in Alaska and northwest Canada. The high latitude sampling locations used in this study are all geographic regions that have not experienced any direct supplementation from translocated or captive bred individuals [97]. Similarly, the contemporary sampling locations from Alaska, northern Canada and Greenland do not differ in allele frequencies or levels of genetic diversity when compared to pre-decline historic samples from Canada ([35]; data not shown) suggesting genetic stability over time at least in their northern distribution. This conclusion is further supported by a significant isolation by distance relationship among breeding sample locations of these two subspecies, even after excluding samples from southern Ontario where introgression from captive-released birds is likely to have occurred (see [35]). A few studies have documented reduced levels of genetic variation in declining populations of birds of prey (e.g., [98]–[100]). However, populations that have experienced recent declines yet have recovered in abundance can retain levels of genetic diversity when certain demographic conditions exist. For example, Hailer et al. [101] documented high levels of genetic diversity in recovering white-tailed eagle (*Haliaeetus albicilla*) populations suggesting that their long life span (∼17 years) has helped buffer against the effects of genetic drift and loss of genetic variability [see also 102–104]. In comparison, maximum longevity of banded wild peregrine falcons ranges from 16 to 20 years [13], suggesting that this factor may have helped populations maintain high levels of genetic variability in their northern distribution in North America, despite significant declines in abundance.

After adjusting significance levels to account for multiple simultaneous comparisons, five sample locations possessed significant *F*_IS_ values, or heterozygote deficiencies (Table 1). Possible explanations for these results include null alleles, sampled multiple populations (Wahlund effect), or nonrandom mating within sampled locations. Two of the populations were no longer significant after excluding locus Fp92-1 from the analysis, suggesting possible null alleles with this locus. For the three remaining sampled locations, despite observing little population genetic differentiation (*F*_ST_, *D*_EST_, Structure, TESS) between breeding areas across their North American high latitude distribution, the more plausible explanation for significant *F*_IS_ values is due to sampling multiple subpopulations within the three areas (1990 & 2001-04 Greenland *F. p. tundrius* and Northwest *F. p. anatum*). For example, the samples for Greenland 2001-04 were obtained from both Thule (n = 15) and Kangerlussuaq (n = 27), two geographic areas separated by >1,000 km, and the samples from Northwest *F. p. anatum* were obtained from a large geographic area throughout Alaska and northwest Canada [see Figure 1]. Although fewer samples were obtained from Thule, significant *F*_IS_ values were observed, but not with Kangerlussuaq after separating the two datasets (data not shown). More work is required to investigate whether fine scale geographic structure may exist in areas on the periphery of the species' distribution and whether recent expansion or growth may influence these results (e.g., [105]) because peregrine falcons have recently expanded northward into areas such as Thule [33].

We currently do not possess an adequate number of samples collected in the United States to determine if peregrine falcons (i.e., *anatum*) show a similar lack of population breeding structure south of the Canadian and U.S. border (e.g., continental U.S.) and whether contemporary gene flow exists throughout their latitudinal distribution. We feel that this deficiency in sampling, however, does not negate the utility of the current analysis because we were primarily interested in determining overall genetic stability and effective population size of migrant “passage” peregrine falcon “population”. It has been shown that peregrine falcons that breed south of the U.S./Canada border possess reduced migratory behavior than those further north [11], [13], [32] and, therefore, less likely sampled in Padre Island, TX. Although, anecdotal evidence suggests that individuals with mixed *tundrius* ancestry that were released in eastern U.S. may migrate further south than pre-decline individuals (i.e., *anatum*) from the same area [81], other work in the Midwestern U.S. has documented an increasing number of urban-nesting peregrine falcons overwintering consistently in or near their breeding territories [106]. The most recent USFWS Monitoring Results for *F. p. anatum* in 2003 [107] reported that while 92% of recorded nest sites throughout five of the six defined regions for monitoring purposes in continental U.S. were located on natural substrates (e.g., cliffs), 68% in the Midwestern/Northeast region were on human-built structures in urban settings such as tall buildings and bridges [see also 79,106,108,109]. Whether introgression of non-native genes into the breeding population in the U.S. has had any negative effects on fitness (e.g., outbreeding depression; [110]) or changes in population dispersal patterns remains to be shown ([79]; see also [111]). Additional work is required specifically to address the genetic stability of populations in the contiguous U.S., whereas our study is primarily focused on high latitude populations, which are more likely the source of migrant birds passing through Padre Island.

### Migrant population genetic stability

Peregrine falcons sampled during migration at Padre Island, Texas clustered with high support with individuals sampled throughout their northern breeding distribution in Alaska, Canada and Greenland (*F. p. tundrius/anatum*; Fig. 2). In contrast to peregrine falcons sampled in southern Canada (*anatum*), the Padre Island samples had consistently higher posterior probability assignment values similar to peregrine falcons identified as *tundrius/anatum* and possessed little if no signal indicating admixture from the other sampled subspecies (Fig. 2). These results, along with results from *F*_ST_ and *D*_EST_ pairwise comparison (Table S1), therefore suggest that peregrine falcons passing through Padre Island were likely individuals with breeding territories located further north than southeastern Canada. Previous studies investigating migratory patterns of peregrine falcons in North America using banding records or satellite telemetry have also documented peregrines passing through Padre Island that originate or finalize their migration in northern high latitude areas rather than further south in southern Canada and continental U.S. ([11], [32], [33]; see also Fig. 2 in [31]). Fuller et al. [12], for example, identified a wide distribution of breeding territories across northern latitudes for passage peregrines (n = 54), including those surveyed migrating through Padre Island.

Across migratory seasons (fall and spring) and seven time periods sampled over a twenty-two year period, no significant changes in levels of microsatellite diversity were observed at Padre Island, and diversity levels were similar to those obtained from high latitude breeding peregrine falcon sample locations (Table 1). These results suggest that the population is large enough in size to offset the negative effects of drift, with adequate levels of gene flow between areas (see also [112]–[115]). Further, no significant levels of differentiation were observed between each of the sampled time periods (Fig. 2; Table S1). The latter result is important because monitoring changes in population differentiation (e.g., *F*_ST_ or *D*_EST_) is often a more sensitive indicator of population decline than is the loss of allelic diversity [116], [117]. Similarly, no significant change in diversity levels (Table 1) or population differentiation (Table S1) was observed between the two temporal sample periods (1990 and 2001-04) in western Greenland. Multiple studies have documented significant allele frequency change associated with increased population differentiation in small or declining populations [74], [118]–[121]. With our simulated datasets of known size, for example, the development of significant population differentiation (*F*_ST_) was observed in all pairwise comparisons at *N*_e_ of ≤300 in as little as seven generations (the time period between our samples collected from Padre). However, at *N*_e_ of 500 only eight of the forty-five comparisons were significant (18%) and none of the comparisons at *N*_e_ of ≥1000 were significant (0%).

These results, along with those from the USFWS nationwide monitoring efforts [107], suggest that the higher latitude migratory and breeding peregrine falcon population is stable with no indication of decline. In fact, monitoring efforts in the field, including multiple long-term migration watchsites (e.g., Cape May Bird Observatory, Hawk Mountain Sanctuary, Hawk Ridge Bird Observatory) suggest that this species continues to increase in abundance [13], [31], [122]–[124]. Similarly, levels of organochlorine pesticides continue to decline in peregrine falcon migrants returning from Central and South America. Henny et al. [125] reported a 96–97% decline in blood DDE concentrations in female peregrine falcons sampled between 1994 (n = 45) and 2004 (n = 27) at Padre Island. Out of the 27 adult peregrine falcons sampled in 2004, DDE concentrations were below detectible levels (<0.02 µg/g) in 20 birds (77%), while in contrast only two of the 156 adult samples (1%) between 1978 and 1994 were below the detectible limit [125]. These are definitely reassuring signs that the peregrine falcon population is moving toward full recovery.

Our inability to obtain a precise estimate of *N*_e_ for the migratory population of peregrine falcons also suggests a large population [see also 126,127]. Estimates of *N*_e_ ranged from 509 to infinity (>10,000; *N*_e-MAX_), with extremely wide 95% confidence intervals (Table 2). The power to estimate *N*_e_ using genetic data is dependent on multiple factors. When populations are of small size (<500 breeding individuals), a variety of methods, some of which were employed in this study, do exceptionally well in inferring how strong genetic drift was or how large the *N*_e_ of the population must be to cause the observed change in allele frequencies over time when assuming no mutation, selection and migration during the sampled time period [58], [64], [127]. When populations are of large size, however, allele frequencies are less likely to change due to drift and our ability to estimate *N*_e_ becomes much more difficult. As was observed with our estimates of population differentiation measures (*F*_ST_ and *D*_EST_; Table S1) between temporal Padre Island sampling periods, no significant changes in allele frequencies were identified over the 22-year period (∼7 generations), further supporting that the breeding population of high latitude peregrine falcons is of large size. This conclusion is also supported by our estimates of *N*_e_ from the local Greenland peregrine falcon population (*N*_e_ = ∼120) while accounting for immigration, suggesting that a much larger migratory population must exist when extrapolated to the remainder of its breeding distribution across North America.

There are ways to improve the precision of our estimate of *N*_e_. Either we increase the sample size of each time period and/or increase the number of loci characterizing allele frequency change over time. In a recent study investigating *N*_e_ of an Australian tiger prawn (*Penaeus esculentus*) population, Ovenden et al. [128] determined using simulation that they would require 2,000 samples taken one generation apart to reliably estimate *N*_e_ of about 8,000 breeding individuals with eight microsatellite loci using similar temporal methods as this study. By increasing the time between sampling periods to four generations, their simulations suggested that the same sample size would produce accurate estimates for *N*_e_ of 10,000; however, by decreasing the sample size to 1,000 individuals, their ability to obtain finite estimates dropped from 100% to 65% [128]. In this study, our samples sizes were 46 individuals for the majority of temporal periods. We could possibly double the sample size to approximately 100 individuals per time period, but sampling beyond that number is unrealistic given the difficulty in trapping migratory falcons. Palstra & Ruzzante [64] recommend that the *S*/*N*_e_ ratio (where *S* is the sample size) be approximately 0.10 for adequate sampling for the temporal approach for estimating *N*_e_ with genetic data. With a sample size of 46 individuals, we should be able to provide reliable estimates of *N*_e_∼460. Therefore, the wide 95% confidence intervals, or low precision, we obtained from all of the temporal methods for estimating *N*_e_ in this study suggests that the actual *N*_e_ is of larger size.

Alternatively, increasing the number of loci would also increase the power to estimate *N*_e_ from large populations using genetic data; however, the extent of its improvement will depend on the variability of the loci with increased polymorphism required [64]. In a recent review, Leberg [1] commented that ‘increasing the number of loci sampled will increase the precision of estimates to a greater extent than increasing the number of individuals sampled.’ (but see [127]). Therefore, because of difficulties obtaining large sample sizes, it may prove worthwhile to explore additional markers, such as SNPs [e.g., 129–131] and additional microsatellite loci [e.g., 132,133] to obtain a more precise estimate of *N*_e_ that can be used to make future management decisions for this species.

### Conservation implications

The above results can inform future management decisions impacting the full recovery of peregrine falcons breeding in North America. Recently, the U.S. Fish and Wildlife Service allowed the take in 2009 of up to 36 first-year (FY) autumn migrant “passage” peregrine falcons east of 100°W longitude for use in falconry [31], [134]. The plan made a distinction between peregrines with natal sites south of 54°N latitude (*F. p. anatum*) and those further north, which includes both *F. p. tundrius* and northern *F. p. anatum* subspecies. Peregrine falcons (*F. p. anatum*) south of 54°N latitude possess reduced migratory behavior compared to those further north, producing a “leap-frog” breeding/migratory peregrine distribution [11], [13], [31], [32]. This distinction between northern migratory birds and those further south was important because the two geographic groups differ in their current census estimates. Approximately 2,700 to 8,000 pairs, which include both *tundrius* and *anatum* subspecies, are estimated for the northern population (≥54°N latitude) based on non-genetic methods. The southern populations (<54 °N latitude) east of 100°W longitude (*F. p. anatum*) is estimated at ∼450 pairs, while west of 100°W longitude (*F. p. anatum* and *F. p. pealei*) consist of ∼1,400 to 1,800 pairs [31]. Therefore, the USFWS specifically targets the take of migratory individuals that breed ≥54°N latitude.

Based on our genetic results, including census estimates from the field, and because the proposal specified autumn first-year individuals, the removal of 36 FY autumn migrants from the population is unlikely to adversely affect their continued recovery [see also 31]. First-year survivorship in the wild is estimated at 40–50%, while breeding adult survival for migrants is likely between 80–85% [13]. If the peregrine falcon population was of small size (*N*_e_<500), the methods that we employed should have had sufficient power to provide precise estimates of *N*_e_; yet, our inability to obtain such an estimate suggests this population is of larger size. The estimate of *N*_e_ as measured in this study (with the exception of *N*_e_ from LDNe; see [1], [73]) reflects the harmonic mean effective size of the migratory high latitude peregrine falcon population over the sampled seven-generation time period (e.g., 1989–2007). It roughly approximates to the number of breeding individuals that produce offspring that live to reproductive age during the sampled time period, which is typically a value much smaller than the actual census size (*N*) of the population (*N*_e_/*N*∼0.11; see [135]). Genetic measures of *N*_e_ incorporate all demographic effects in their estimate, such as fluctuating population size, unequal sex ratio, and variance in reproductive success [1], [136]–[138], which all decrease *N*_e_ relative to *N*.

Therefore, even if we assume that *N*_e_ of 500 is correct, the actual census size is likely an order of magnitude larger; however, more work is required to determine the actual ratio between the two measures specific to peregrine falcon populations. The number of non-breeders (floaters) can outnumber the actual breeders in some areas by severalfold [13], [139], and the numbers of migrants recorded at specific monitoring sites in the U.S. are high. For example, between 1999 and 2004, the mean annual fall migration peregrine falcon count at Curry Hammock Florida State Park in the middle Keys alone was 1,908 individuals (1,432–2,858, min-max; [122]). Taking into account the relatively high first year mortality for migrant peregrine falcons (≥50–60%, [13]), the *N*_e_/*N* ratio is further reduced due to a large number of individuals not producing offspring relative to fall census estimates.

Although we were unable to provide a precise point estimate of *N*_e_ in this study, we can conclude that the *N*_e_ for the migrant peregrine falcon population is unlikely to be smaller than 500. This agrees with field data suggesting a much larger breeding population size [13], [31]. Reducing organochlorine pesticides and other contaminants (e.g., mercury; [140], [141]) in their environment is of greater importance for securing the long-term viability of peregrine falcon populations, and recent results from Henny et al. [125] suggest that these conditions are improving. Consequences of the illegal take of peregrine falcons in their wintering distribution in South America [142] also deserves more attention, and certainly, local monitoring of specific areas within the species' breeding distribution should continue. With the exception of our results from Greenland, the analyses using migrant samples from Padre Island provide a coarse description of population genetic stability over time for the high-latitude breeding population of this species, while more local demographic perturbations associated with specific breeding locations (see [143]) would not necessarily be reflected in our results using migrant samples alone.

## Supporting Information

Table S1 Pairwise estimates of *F*_ST_ (below diagonal) and *D*_est_ (above diagonal) based on 11 microsatellite loci between regional peregrine falcon sample locations.
(0.09 MB PDF)
