# Supplementary material for: The Use of Genetics for the Management of a Recovering Population: Temporal Assessment of Migratory Peregrine Falcons in North America
Source: PLoS One. 2010 Nov 18;5(11):e14042. doi: 10.1371/journal.pone.0014042 (PMC2987794; doi:10.1371/journal.pone.0014042)
Supplement: Table S1 — Pairwise estimates of F ST (below diagonal) and D est (above diagonal) based on 11 microsatellite loci between regional peregrine falcon sample locations. (0.09 MB PDF) [file pone.0014042.s001.pdf]

Table S1. Pairwise estimates of F_{ST} (below diagonal) and D_{est} (above diagonal) based on 11 microsatellite loci between regional peregrine falcon sample locations.

		F. p. pealei	*F. p. tundrius*					*F. p. anatum*			
			Northwest	Nunavut	Ungava Bay	Greenland	Greenland_90[1]	Northwest	Alberta	Ontario	Northeast
F. p. pealei	Pacific Northwest	--	***0.095***	***0.077***	***0.097***	***0.069***	***0.071***	***0.075***	***0.057***	***0.028***	***0.068***
F. p. tundrius	Northwest	***0.099***	--	0.003	-0.020	0.009	0.012	-0.008	0.003	0.032	0.015
	Nunavut	***0.076***	0.016	--	-0.001	-0.003	0.012	0.020	0.000	0.014	0.026
	Ungava Bay	***0.110***	0.010	0.019	--	-0.002	-0.001	0.013	0.022	0.030	0.031
	Greenland	***0.070***	***0.023***	0.007	0.019	--	-0.001	0.015	0.002	0.014	0.025
	Greenland_90[1]	***0.071***	***0.026***	***0.020***	0.020	0.008	--	0.009	0.006	***0.026***	0.030
F. p. anatum	Northwest	***0.080***	0.006	***0.025***	***0.033***	***0.022***	***0.018***	--	0.008	***0.029***	0.018
	Alberta	***0.067***	0.022	0.014	***0.045***	***0.017***	***0.020***	***0.019***	--	0.003	0.022
	Ontario	***0.036***	***0.041***	***0.021***	***0.046***	***0.021***	***0.031***	***0.033***	0.016	--	0.013
	Northeast	***0.074***	0.032	***0.034***	***0.050***	***0.034***	***0.039***	***0.029***	***0.035***	***0.024***	--
F. p. cassini	Argentina	***0.266***	***0.239***	***0.196***	***0.242***	***0.199***	***0.208***	***0.191***	***0.199***	***0.196***	***0.215***
F. p. macropus	Australia	***0.293***	***0.318***	***0.285***	***0.338***	***0.302***	***0.281***	***0.300***	***0.323***	***0.285***	***0.355***
F. p. tundrius/	Fall 1985	***0.076***	0.018	0.005	0.025	0.006	0.015	***0.027***	0.016	***0.028***	***0.042***
anatum	Spg 1986	***0.069***	0.011	0.003	0.021	0.001	***0.013***	***0.018***	0.012	***0.016***	***0.027***
(Padre Island)	Fall 1988	***0.090***	0.017	0.008	***0.029***	0.002	***0.016***	***0.022***	0.013	***0.033***	***0.044***
	Spg 1989	***0.059***	0.011	0.005	***0.027***	0.004	0.014	***0.017***	0.008	***0.013***	***0.027***
	Spg 2001	***0.087***	0.017	0.003	0.024	-0.001	***0.011***	***0.021***	0.013	***0.029***	***0.034***
	Fall 2006	***0.089***	***0.019***	0.003	***0.030***	0.004	***0.018***	***0.026***	0.016	***0.029***	***0.032***
	Spg 2007	***0.095***	0.009	0.005	0.013	0.000	0.012	0.012	0.015	***0.026***	***0.032***

Table S1 (*continued*)

		F. p. *cassini*	*F. p.* *macropus*	*F. p. tundrius/anatum* - Padre Island migrants						
				Fall 1985	Spg 1986	Fall 1988	Spg 1989	Spg 2001	Fall 2006	Spg 2007
F. p. pealei	Pacific Northwest	***0.293***	***0.273***	***0.074***	***0.068***	***0.093***	***0.054***	***0.084***	***0.091***	***0.089***
F. p. tundrius	Northwest	***0.255***	***0.310***	0.004	-0.004	0.002	-0.003	0.003	0.004	-0.008
	Nunavut	***0.228***	***0.315***	-0.005	-0.007	-0.002	-0.004	-0.005	-0.008	-0.007
	Ungava Bay	***0.222***	***0.284***	0.004	0.003	0.010	0.007	0.002	0.009	-0.013
	Greenland	***0.218***	***0.319***	-0.003	-0.008	-0.009	-0.006	-0.011	-0.006	-0.014
	Greenland_90	***0.234***	***0.293***	0.006	0.003	0.007	0.004	0.002	0.009	-0.001
F. p. anatum	Northwest	***0.210***	***0.323***	***0.022***	0.012	0.016	0.011	0.017	0.018	0.002
	Alberta	***0.207***	***0.332***	0.003	-0.002	-0.001	-0.007	0.001	0.001	-0.002
	Ontario	***0.225***	***0.310***	***0.023***	0.009	***0.029***	0.005	***0.026***	0.023	0.017
	Northeast	***0.235***	***0.397***	0.035	0.017	0.037	0.016	0.026	0.021	0.019
F. p. cassini	Argentina	--	***0.550***	0.273	0.231	0.234	0.271	0.218	0.222	0.184
F. p. macropus	Australia	***0.521***	--	0.304	0.312	0.325	0.316	0.330	0.316	0.324
F. p. tundrius/	Fall 1985	***0.235***	***0.289***	--	-0.007	-0.009	-0.006	-0.009	-0.008	-0.003
anatum	Spg 1986	***0.198***	***0.284***	0.003	--	-0.009	-0.013	-0.012	-0.010	-0.010
(Padre Island)	Fall 1988	***0.205***	***0.299***	0.001	0.001	--	-0.006	-0.015	-0.015	-0.009
	Spg 1989	***0.229***	***0.292***	0.003	-0.002	0.004	--	-0.005	-0.006	-0.003
	Spg 2001	***0.207***	***0.320***	0.000	-0.004	-0.007	0.002	--	-0.015	-0.012
	Fall 2006	***0.205***	***0.304***	0.002	0.001	-0.004	0.005	-0.005	--	-0.012
	Spg 2007	***0.188***	***0.325***	0.009	0.001	0.002	0.008	0.000	0.001	--

Significant F_{ST} (corrected for multiple comparisons) and D_{est} (95% CI that do not include zero) values are indicated in bold italics. Negative (-) values indicate a differentiation value near zero (0.000).

[1]Greenland samples collected in 1990
